# Complete Genome Sequence of Fish Pathogen Flavobacterium columnare Strain B185, Originating from Finland

**DOI:** 10.1128/MRA.01285-19

**Published:** 2019-12-05

**Authors:** Janne J. Ravantti, Elina Laanto, Petri Papponen, Lotta-Riina Sundberg

**Affiliations:** a Molecular and Integrative Biosciences Research Programme, Faculty of Biological and Environmental Sciences, University of Helsinki, Helsinki, Finland; b Applied Tumor Genomics Research Program & Department of Medical Genetics, University of Helsinki, Helsinki, Finland; c Department of Biological and Environmental Science and Nanoscience Center, University of Jyvaskyla, Jyvaskyla, Finland; University of Maryland School of Medicine

## Abstract

We report a complete genome sequence of a Finnish isolate of the fish pathogen Flavobacterium columnare. Using PacBio RS II sequencing technology, the complete circular genome of *F. columnare* strain B185 with 3,261,404 bp was obtained.

## ANNOUNCEMENT

Flavobacterium columnare (*Bacteroidetes*) is an opportunistic fish pathogen affecting freshwater fish farming worldwide. As of January 2019, fewer than 20 genomes of *F. columnare* were available, and 5 were complete. We report a whole-genome sequence of *F. columnare* strain B185, isolated from Finland. *F. columnare* isolates have been traditionally divided into different genetic groups, and recently, four groups were assigned (1). All described Finnish isolates belonging to the genetic group I and have been further divided into five genotypes (A to G) (2), and strain B185 represents genotype G. Strain B185 has been used in several studies and confirmed as virulent (3, 4). This genome sequence will aid in resolving the genetics of virulence in *F. columnare* and in deciphering the genetic diversity and evolutionary origins of Finnish *F. columnare* strains.

Flavobacterium columnare strain B185 was isolated in 2008 from tank water at a fish farm in Central Finland during antibiotic treatment in a quarantine unit. The water sample was plated onto Shieh agar, and an *F. columnare* colony was isolated, purified, and further cultivated in Shieh liquid medium and stored at –80°C. Genomic DNA was extracted from an overnight (O/N) culture using a DNeasy blood and tissue kit (Qiagen) according to the manufacturer’s instructions for Gram-negative cells. DNA was sequenced at the Institute of Biotechnology, University of Helsinki, Helsinki, Finland, using PacBio RS II technology with P4-C2 sequencing chemistry, generating 268,103 reads and an average read length of 3,962 bp with an *N*_50_ value of 6,100 bp. After the genome was assembled with the HGAP3 (SMRT portal) (5), a single contig remained. The Gap4 (6) program was then used to confirm that both ends contained overlapping sequence, indicating a circular genome. The overlaps were merged, and finally, the genome was rotated so that the starting point was at the gene *dnaA*. The final assembly yielded one scaffold with an average sequence coverage of 224.6×. The genome of *F. columnare* strain B185 consists of 3,261,404 bp with a GC content of 31.7%. No plasmids were detected. A total of 2,812 coding DNA sequences (CDSs) were predicted using NCBI’s Prokaryotic Genome Annotation Pipeline (7). In all the analyses, default parameters were used except where otherwise noted. The genome contains 3 rRNA operons and 92 tRNA genes. Amino acid-level similarities (global alignment per translated open reading frames [ORFs] with 80% similarity cutoff) to other complete *F. columnare* genomes from genomic group I were 92% to strain TC 1691, 91% to strain Pf1, and 90% to strain ATCC 49512 and from genomic group II were 81% to strain C#2 and 80% to strain 94-081. Two functional CRISPR loci (type II-C and type VI-B) were identified earlier in an *F. columnare* genome (8) and are also present in strain B185. Interestingly, the II-C locus has a putative parDE type II toxin-antitoxin (TA) system between the *cas9* and the repeat region. A putative prophage of approximately 30 kbp in length was detected with HHPred (9) analysis for regions of annotated hypothetical proteins. Gene modules, e.g., for head and tail morphogenesis, were identified. In addition, we identified a putative ∼40-kb genetic island rich in integrases and transposases.

### Data availability.

The complete genome sequence of Flavobacterium columnare strain B185 has been deposited in GenBank under the accession number NZ_CP010992, and the raw sequence data were submitted to the SRA under accession number PRJNA276168.
